# The Deep Past of the White Shark, *Carcharodon carcharias*, in the Mediterranean Sea: A Synthesis of Its Palaeobiology and Palaeoecology

**DOI:** 10.3390/life13102085

**Published:** 2023-10-20

**Authors:** Alberto Collareta, Simone Casati, Andrea Di Cencio, Giovanni Bianucci

**Affiliations:** 1 Dipartimento di Scienze della Terra, Università di Pisa, Via S. Maria 53, 56126 Pisa, PI, Italy; alberto.collareta@unipi.it; 2 Museo di Storia Naturale, Università di Pisa, Via Roma 79, 56011 Calci, PI, Italy; 3 Gruppo Avis Mineralogia e Paleontologia Scandicci, Piazza Vittorio Veneto 1, Badia a Settimo, 50018 Scandicci, FI, Italy; sim.casati@gmail.com (S.C.); andrea.dicencio@gmail.com (A.D.C.); 4 Istituto Comprensivo “Vasco Pratolini”, Via G. Marconi 11, 50018 Scandicci, FI, Italy; 5 Studio Tecnico Geologia e Paleontologia, Via Fratelli Rosselli 4, 50026 San Casciano Val di Pesa, FI, Italy

**Keywords:** conservation palaeobiology, Elasmobranchii, fossil record, Lamnidae, palaeoichthyology, Pleistocene, Pliocene, Quaternary, vertebrate palaeontology, white pointer

## Abstract

The white shark, *Carcharodon carcharias*, is the main top predator of the present-day Mediterranean Sea. The deep past of *C. carcharias* in the Mediterranean is witnessed by a rather conspicuous, mostly Pliocene fossil record. Here, we provide a synthesis of the palaeobiology and palaeoecology of the Mediterranean white sharks. Phenetically modern white shark teeth first appeared around the Miocene–Pliocene transition in the Pacific, and soon after in the Mediterranean. Molecular phylogenetic analyses support an origin of the Mediterranean white shark population from the dispersal of Australian/Pacific palaeopopulations, which may have occurred through the Central American Seaway. Tooth dimensions suggest that the Mediterranean white sharks could have grown up to about 7 m total length during the Pliocene. A richer-than-today marine mammal fauna was likely pivotal in supporting the Mediterranean white sharks through the Pliocene and most of the Quaternary. White sharks have seemingly become more common as other macropredators declined and disappeared, notwithstanding the concurrent demise of many potential prey items in the context of the latest Pliocene and Quaternary climatic and environmental perturbations of the Mediterranean region. The overall generalist trophic habits of *C. carcharias* were likely crucial for securing ecological success in the highly variable Mediterranean scenario by allowing the transition to a mostly piscivorous diet as the regional marine mammal fauna shrank.

## 1. Introduction and Rationale

The white shark, *Carcharodon carcharias* (Elasmobranchii: Lamnidae), is widespread in the warm to cool temperate quarters of the global ocean, including the Mediterranean Sea [1,2,3,4,5]. In spite of a long history of sightings, however, the Mediterranean white shark population is still rather poorly known in terms of provenance [6] and ecology [7]. That said, *C. carcharias* is generally considered to be the unrivalled elasmobranch apex predator of the Mediterranean Sea [8], as it is worldwide [9]. More specifically, recent research suggests that the Mediterranean white sharks are tertiary piscivores that feed at a significantly higher trophic level than other co-occurring macropredatory elasmobranchs such as *Hexanchus griseus* and *Isurus oxyrinchus* [10], but episodes of predation and scavenging on marine mammals have also been observed and/or inferred from stomach content analyses [11,12,13]. *Carcharodon carcharias* is rare in the present-day Mediterranean Basin [14], being relatively more common in the Adriatic Sea, Sicilian Channel and Tyrrhenian Sea [7,15]. Some marine areas along the Tunisian and Sicilian coasts [16,17,18], as well as in the Aegean Sea [19,20], have been proposed to serve as nursery grounds for the Mediterranean white sharks.

**Figure 1:**
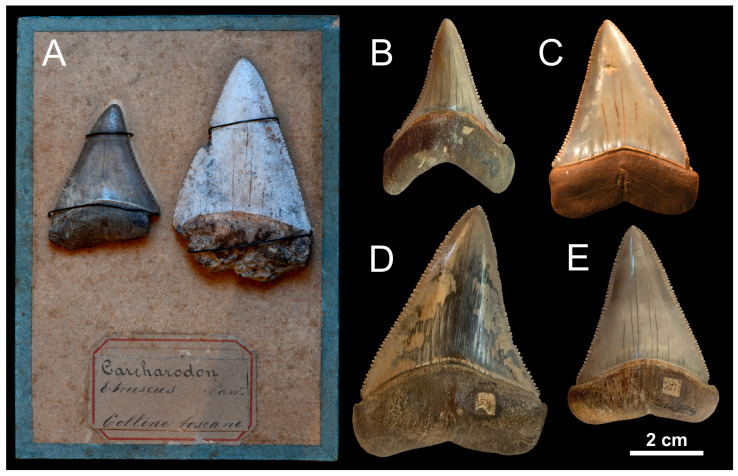
Pliocene fossils of *Carcharodon carcharias* from the Mediterranean Basin. (**A**) Two teeth from the “Tuscan hills” (Tuscany, central Italy), stored in the Collezione di Geologia “Museo Giovanni Capellini” (=MGGC, Bologna), with their original supporting tablet and historical label. (**B**) Tooth from Peccioli (Tuscany), stored in the Museo di Storia Naturale dell’Università di Pisa (=MSNUP, Calci). (**C**) Tooth from Orciano Pisano (Tuscany), stored in the Museo Geopaleontologico Scienze della Terra del “Gruppo AVIS Mineralogia e Paleontologia Scandicci” (=GAMPS, Badia a Settimo, Scandicci). (**D**) Tooth from Peccioli, stored in the MSNUP. (**E**) Tooth from the surroundings of Piacenza (Emilia-Romagna, northern Italy), stored in the MSNUP. Specimen codes are as follows: “Tavoletta n. 18 quarto” (**A**); MSNUP I12972 (**B**); GAMPS-00875 (**C**); MSNUP I12979 (**D**); MSNUP I12735 (panel **E**). All specimens are depicted in lingual view.

**Figure 2:**
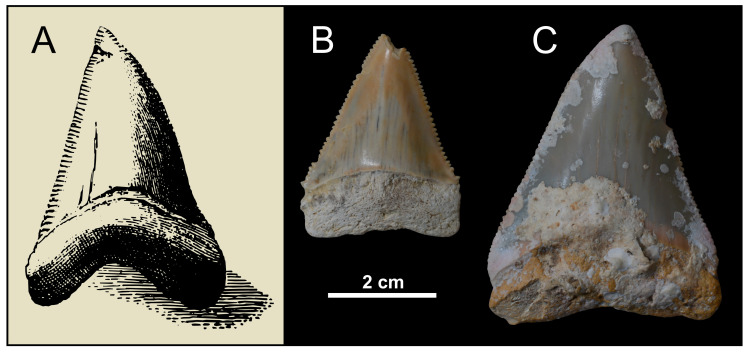
Quaternary fossils of *Carcharodon carcharias* from the Mediterranean Basin. (**A**) Excerpt of Armstrong’s [23] plate IV, depicting a white shark tooth from Menorca (Balearic Islands, Mediterranean Spain). (**B**) Tooth from Fauglia (Tuscany, central Italy), stored in the Museo Geopaleontologico Scienze della Terra del “Gruppo AVIS Mineralogia e Paleontologia Scandicci” (=GAMPS, Badia a Settimo-Scandicci). (**C**) Tooth from the Meloria shoals (Tuscany, central Italy), stored in the GAMPS. Although some issues exist about the stratigraphic provenance of the specimens depicted in (**A**,**C**), they are likely Pleistocene in age [22,24]. Specimen codes are as follows: GAMPS-01073 (**B**); GAMPS-01072 (**C**). Note that the scale bar only applies to (**B**,**C**). All specimens are depicted in lingual view.

**Figure 3:**
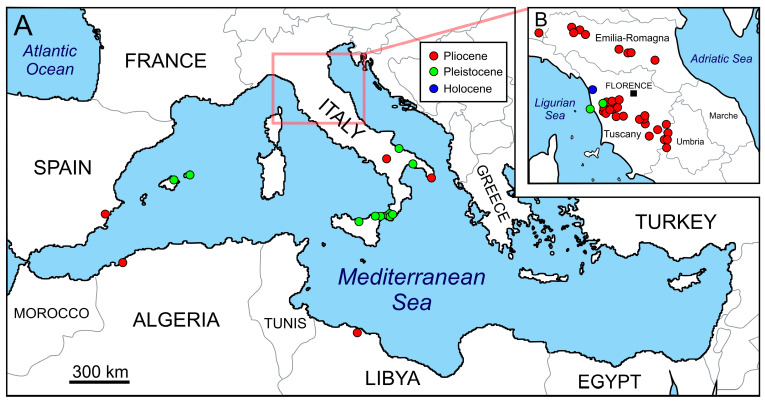
Chronostratigraphic and geographic distribution of fossil records of *Carcharodon carcharias* from the peri-Mediterranean area (**A**), with a close-up of central and northern Italy, where most finds concentrate (**B**). Data after the many works by Vicens and Gracia [25], Bianucci et al. [11], Bisconti [26], Dominici et al. [27], Adnet et al. [28], Pawellek et al. [29], Danise and Dominici [30], Freschi [31], Bendella et al. [32], Bernárdez and Rábano [24], Zazzera et al. [33] and Collareta et al. [22]. Note that records that originate from outside the Mediterranean (palaeo)geographic domain (e.g., from the Guadalquivir Basin of southern Spain; [34]) are not shown in the figure. The position of the Pliocene–Pleistocene boundary follows Gibbard et al. [35].

Nowadays, the Mediterranean white sharks—which the IUCN Red List classifies as critically endangered [21]—appear to be declining regionally as a possible consequence of the progressive anthropization of the Mediterranean ecosystems [5,7]. In this context, understanding how the Mediterranean population of *C. carcharias* looked like well before any human impact may prove important [22]. Luckily, the deep past of *C. carcharias* in the Mediterranean Basin is witnessed by a rather abundant record of fossil teeth, with many finds being Pliocene (ca. 5.3–2.6 Ma) in age (Figure 1), though a growing number of geologically younger finds also exist (Figure 2). Most of these finds come from several localities of Italy, where abundant coastal and shelfal sediments were deposited at the foot of the Apennines and subsequently uplifted to outcrop (Figure 3).

Here, we provide a synthesis of the palaeobiology and palaeoecology of *C. carcharias* in the Mediterranean Basin from the earliest part of the Pliocene onwards. Such an effort builds upon an extensive review of the relevant palaeoichthyological literature, most of which has been summarised and referenced by Marsili [36], as well as on some selected neontological papers. Rather than pursuing a methodical approach to the topic, the present paper is organised as a commentary around four main questions, namely, what do the fossil record and the molecular data disclose about the origin and early history of the Mediterranean population of white sharks? What can palaeontology reveal about the maximum body size of *C. carcharias* in the Mediterranean realm? What does it tell us about the trophic ecology of the ancient Mediterranean white sharks? How did *C. carcharias* become the main Mediterranean top predator?

Each of the following paragraphs will try and provide some palaeontologically grounded answers to the above research questions.

## 2. Origin of *Carcharodon carcharias* and Its Early History in the Mediterranean Sea

As outlined by Gubili et al. [6], the provenance of the Mediterranean population of *C. carcharias* is both a scientific conundrum and a conservation issue—one that is inextricably linked to the very origin of *C. carcharias* itself. Fiercely debated until the early XXI century, the evolutionary relationships of *C. carcharias* appear to be sufficiently clear at present, as are also the mode and tempo of its appearance in the fossil record. Indeed, evidence now exists of a progressive transition between unserrated teeth belonging to the extinct *Cosmopolitodus* stock of “broad-toothed makos” and typical white shark teeth through variably serrated teeth that occur in Late Miocene deposits of the Pacific Ocean (mostly Chile, Peru, California and Japan) [37,38,39,40,41,42,43]. The recent description of *Carcharodon hubbelli* from the Messinian of Peru provides us with a glimpse of the early evolutionary history of *Carcharodon*; its type specimen includes an articulated dentition, consisting of 222 teeth, which exhibits a mosaic of archaic and modern traits, such as weakly serrated cutting edges and a distally inclined third upper anterior (A3, intermediate) tooth [42,43]. Specimens from the upper portion of the Paraná Formation of Argentina indicate that archaic representatives of *Carcharodon* had reached the south-eastern Atlantic Ocean before the end of the Miocene [44]. The first teeth that fully conform to the living species *C. carcharias* (e.g., by displaying fully developed serrations) date back to about the Miocene–Pliocene boundary [41,45].

Molecular phylogenetic analyses support an origin of the Mediterranean white shark lineage from the eastward dispersal of Australian/Pacific palaeopopulations [46]. Furthermore, Leone et al.’s [46] fossil-calibrated molecular clock suggests that the Mediterranean white shark population separated from other ancestral populations around 3.23 Ma, during the early Piacenzian, after the formation the Isthmus of Panama and the consequent closure of the Central American Seaway (but, see [47] for a later dating of the Panamanian uplift). Commenting on these figures, Leone et al. [46] hypothesised that *C. carcharias* colonised the Mediterranean Sea to occupy the ecological niches left empty by the demise of other marine apex predators, e.g., the giant megatooth shark *Carcharocles megalodon* (also known as *Otodus megalodon*), whose chronostratigraphic range embraces most of the Miocene and Pliocene until ca. 3.5 Ma ([45]; but see also [48] for an alternative extinction age of about 2.6 Ma).

Actually, teeth of *C. carcharias* and *C. megalodon* co-occur within a deposit that locally marks the base of the Pliocene succession of the Sabratah Basin of northwestern Libya [29]. Furthermore, evidence for the occurrence of *C. carcharias* in the early Pliocene Mediterranean Sea comes from southeastern Spain ([28]; records dated at ~5.3–4.19 Ma according to [49]) and, possibly, central Italy ([30]; record dated at 4.62–4.55 Ma according to [50]). These data do not only indicate that *C. carcharias* reached the Euro-Mediterranean region soon after its origin in the Pacific Ocean; they also suggest that white sharks may have colonised the Mediterranean Basin before settling along the Northwestern Atlantic coast, where the appearance of *C. carcharias* appears to have been somewhat delayed [45]. That said, whether *C. carcharias* entered the Mediterranean Basin in occurrence of or slightly subsequent to the widespread restoration of fully marine conditions at the end of the “Messinian Salinity Crisis” ([51], and the many references therein) cannot be said for certain.

## 3. On the Maximum Body Size of the White Shark: Hints from the Mediterranean Fossils

The maximum body size attained by *C. carcharias* represents a vexata quaestio in ichthyological research. Exaggerated claims of extant white sharks as long as 12 m or more have long been debunked [52], and the same can be said for an allegedly 6.4-m-long specimen of *C. carcharias* that was taken around 1943 in Cuba [53,54]. Thus, the largest individuals of *C. carcharias* to have ever been reliably measured account for total length values of 594 cm (a female from Ledge Point, Australia; [53]) and 589 cm (a female from the Gulf of Lion, Mediterranean France; [55]). A reappraisal of the photographic evidence of the largest white sharks captured in the Mediterranean Sea led De Maddalena et al. [56] to infer that “*C. carcharias* can, in rare and exceptional cases, exceed 6 m in length, reaching at least 640–660 cm […] and very probably even more”. Few scientists have, however, embraced these estimates; thus, the recent review by Castro [54] concluded that “[i]f white sharks measuring 6.1 m exist today, they must be specimens at the maximum of their allotted life spans. The chances of such shark surviving in coastal waters or oceans for decades, without encountering the ubiquitous gillnets or longlines, must be very low. We still await some reliable observer who can attest that he or she measured a white shark at 6 m or larger and provide sufficient proof of the measurement”. Similarly, Compagno et al. [2] reported the maximum size of *C. carcharias* as approaching 600 cm in total length.

Various equations have been proposed in the literature which attempt to estimate the body length of *C. carcharias* based on the dimensions of some selected tooth positions, and most notably the Tooth Enameloid Height (hereinafter, TEH) of the largest teeth in the dentition, i.e., the first and second upper anteriors [28,57,58]. This allows for reconstructing the body size of some conspicuous specimens of *C. carcharias* from the Mediterranean Pliocene. Specifically, by applying Shimada’s [58] linear equation for the first and second upper anteriors, a large tooth from the Lower Pliocene of southeastern Spain (TEH = 56 mm; [28]: Figure 2.1) would correspond to a total length of 662 cm, whereas an even larger specimen from the Italian Pliocene (TEH = 59 mm; [59]: pp. 22, 24) would indicate a white shark individual as long as at least 693 cm. Similarly, slightly shorter teeth such as the Italian Pliocene specimen figured by De Stefano ([60]: pl. 2, Figure 5; TEH = 52 mm) would still correspond to a total length of at least 615 cm, that is, larger than the largest verified extant specimen of *C. carcharias* worldwide. Other teeth in the same size range as the aforementioned (TEH between 52 and 59 mm) or even larger are present in palaeontological collections that preserve Mediterranean Pliocene fossils ([61]: Figure 48) (Figure 1D), thus evoking total length values longer than 6 m.

It has been noted, however, that tooth morphology is fairly variable among extant white sharks, with some individuals having long, narrow teeth, whereas others have shorter, wider teeth, such that precise total length calculations based on tooth dimensions have sometimes been met with scepticism [54]. That said, the maximum values of TEH shown by extant white shark teeth seem to fall short of the largest Mediterranean Pliocene examples, which in turn is strongly suggestive of the past occurrence of white shark individuals of unparalleled body size. Indeed, the largest extant white shark tooth we are aware of was reported by Cappo [62], who mentioned a maximum TEH value of 51.6 mm for a very large—but not measured—*C. carcharias* individual whose head had been collected in 1987 off Kangaroo Island, Australia. As for the largest individual of *C. carcharias* to have ever been reliably measured, i.e., the 594 cm long female from Ledge Point, it displays a maximum TEH value of 51 mm [58]. Of the 132 modern white shark specimens that comprise the database behind Adnet et al.’s ([27]: Figure 3) analysis, which gathers measurements from several earlier works, none seem to reach TEH values greater than De Stefano’s [60] largest fossil specimen.

To summarise, the various Mediterranean Pliocene teeth that display TEH > 52 mm appear to have no modern counterparts. As such, they are strongly reminiscent of the past occurrence of white shark individuals larger than the maximum total length values that are commonly cited in recent literature for extant *C. carcharias* worldwide (around 6.0 m). As already highlighted elsewhere [28], the largest among these fossils are suggestive of outstanding body size values, about 7 m in total length. That said, such an apparent decrease in body size since the Pliocene should be approached with caution [28]. Indeed, the mean total length of the Mediterranean white sharks is suspected to have been decreasing since the early XX century, possibly as a consequence of unsustainable fishing pressure [5], such that *C. carcharias* may have grown larger than today before the Mediterranean Basin became strongly anthropized.

## 4. Trophic Ecology of the Long-Gone Mediterranean White Sharks

**Figure 4:**
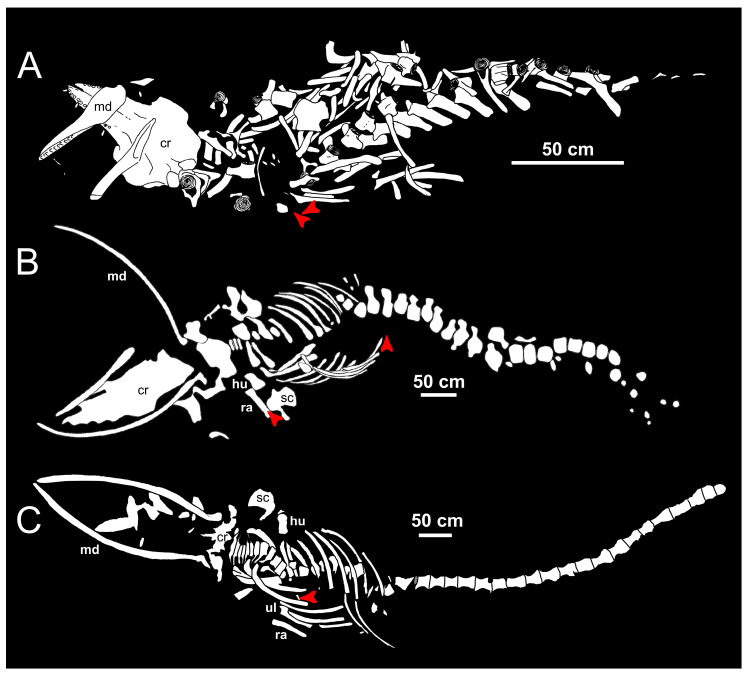
Taphonomic evidence for the trophic ecology of the Mediterranean white sharks in Pliocene times, consisting of marine vertebrate skeletons with associated teeth of *Carcharodon carcharias*. (**A**) Schematic line drawing of a skeleton of *Hemisyntrachelus cortesii* from the Pliocene deposits of Salsomaggiore Terme (Emilia-Romagna, northern Italy). (**B**) Schematic line drawing of a skeleton of Balaenopteridae indet. from the Pliocene deposits of Orciano Pisano (Tuscany, central Italy). (**C**) Schematic line drawing of a skeleton of *Balaenoptera* sp. from the Pleistocene deposits of Lama Lamasinata (Apulia, southern Italy). White shark teeth are displayed in red colour. Anatomical abbreviations are as follows: cr, cranium; hu, humerus; md, mandible; ra, radius; sc, scapula; ul, ulna. (**A**–**C**) redrawn and modified after Bianucci et al. [11], Dominici et al. [27] and Zazzera et al. [33], respectively.

**Figure 5:**
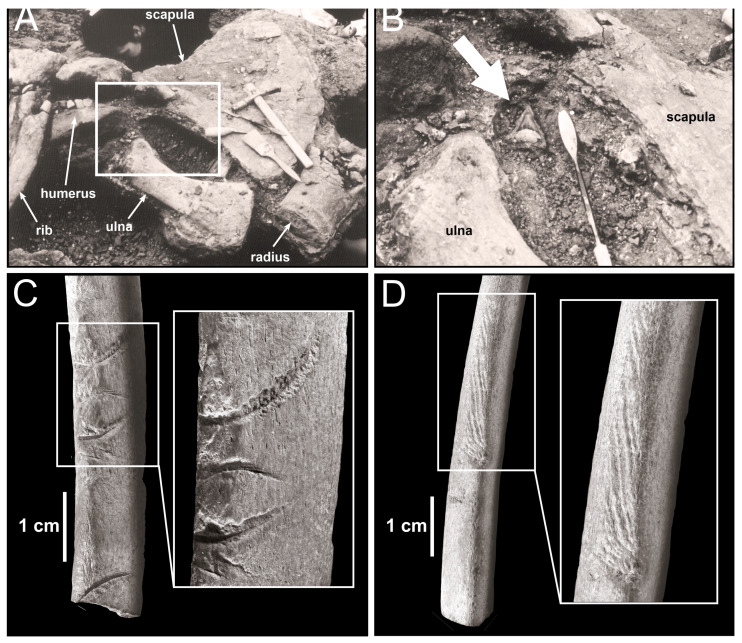
Additional taphonomic evidence for the trophic ecology of the Mediterranean white sharks in Pliocene times. (**A**,**B**) Skeleton of *Balaena* sp. from the Pliocene deposits of Poggio Tagliato (Tuscany, central Italy); close-ups of the disarticulated right forelimb (**A**), and detail thereof showing an associated white shark tooth (indicated by a white arrow) (**B**). (**C**,**D**) Fragmentary cetacean rib from the Pliocene deposits of Buca della Balena (Emilia-Romagna, northern Italy) displaying bite marks attributed to *Carcharodon carcharias*, including characteristically serrated grooves (**C**) and scrapes (**D**). (**C**,**D**) modified after Freschi and Cau ([63]: Figures 5 and 6).

Abundant taphonomic evidence exists of the trophic ecology of the Mediterranean white sharks in Pliocene times ([11] and the many references therein; [31,63]), whereas data about the Pleistocene are essentially limited to a single case study [33]. Consisting of marine vertebrate skeletons with associated teeth of *C. carcharias* (Figure 4 and Figure 5) as well as of marine vertebrate bones that display characteristically serrated bite marks (Figure 5), such evidence indicates that cetaceans used to feature prominently in the trophic spectrum of the Pliocene Mediterranean white sharks. In particular, odontocetes such as *Hemisyntrachelus* (Figure 4A) and other indeterminate delphinids, as well as mysticetes such as *Balaena* and other balaenid right whales (Figure 5A,B), the eschrichtiine grey whale *Eschrichtioides* and several rorqual-like balaenopteroids (Figure 4B,C) were part of the diet of *C. carcharias* in the Pliocene Mediterranean Sea [11]. Evidence for active predation on the relatively common *Hemisyntrachelus* is particularly compelling [59], and this delphinid genus may have represented an important food source for the Mediterranean Pliocene representatives of *C. carcharias*; in turn, size-based considerations suggest that mysticetes were generally consumed as carcasses [11].

Several neontological studies have shown that recent white sharks start preying upon marine mammals such as cetaceans and pinnipeds while entering adulthood, that is, when they reach a body length of 300–400 cm [11,64,65]. That said, as already noted above, the size of some fossil teeth suggests that white sharks about or above 7 m total length wandered the Pliocene Mediterranean Sea. Whether this figure hints at different feeding habits compared to those of extant white sharks is uncertain. One may be led to think that 7-m-long white sharks would have been able to predate large prey items up to about 3–4 m total length. However, isotope investigations on recent and fossil teeth of *C. carcharias* from several sites worldwide (including the aforementioned Libyan locality described by [29]) have suggested that Pliocene white sharks foraged at either the same or a slightly lower trophic level than their extant conspecifics [66,67,68].

**Figure 6:**
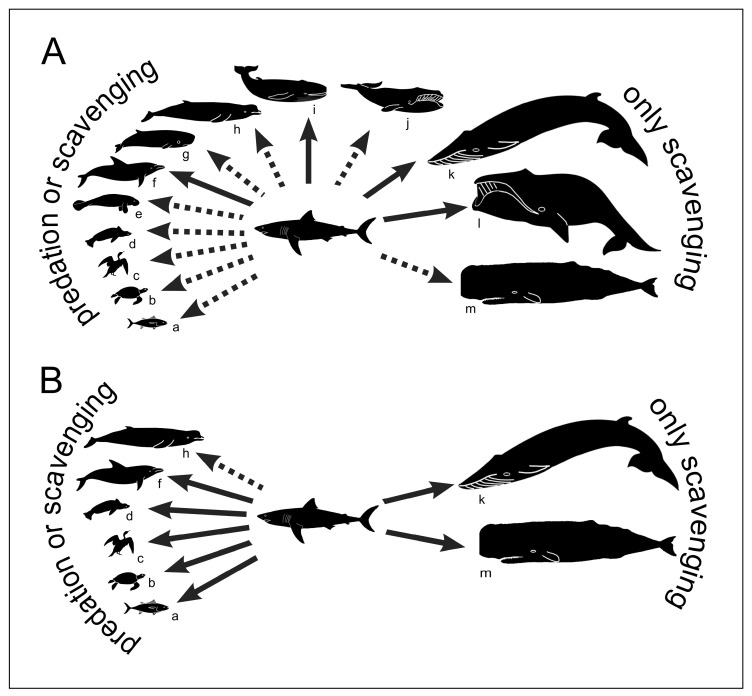
Reconstructed trophic interactions between *Carcharodon carcharias* and other marine vertebrates in the Pliocene (**A**) and present-day (**B**) Mediterranean Sea. Silhouette codes are as follows: a, fishes; b, turtles; c, birds; d, seals; e, sirenians; f, delphinoids (including, in Pliocene times, phocoenids and monodontids besides delphinids); g, kogiids; h, ziphiids; i, small-sized plicogulans (*sensu* [69]); j, small-sized balaenids; k, large-sized plicogulans; l, large-sized balaenids; m, physeterids. Solid arrows indicate trophic interactions that are supported by taphonomic evidence, stomach content analyses and/or direct observations of feeding actions. Dashed arrows indicate hypothetical trophic interactions for which evidence is currently wanting. See the main text for the palaeontological data source; neontological data after Boldrocchi et al. [5]. Both panels modified after Bianucci et al. [11].

As a matter of fact, for most of the Pliocene, the marine mammal stock of the Mediterranean Sea was significantly more diverse than today, including, e.g., representatives of the currently extra Mediterranean cetacean families Balaenidae, Eschrichtiidae (which nowadays are often regarded as a subfamily of Balaenopteridae; [69]), Kogiidae, Monodontidae and Phocoenidae, as well as dugongid sirenians [70,71] (Figure 6). Some of the above groups were represented by morphotypes that have subsequently gone extinct, including small-sized mysticetes (i.e., baleen whales) like *Balaenula* [11,69]; others, such as the sirenians and some cetacean lineages (e.g., the monodontids), have been extirpated from the Mediterranean Sea but survive elsewhere [71,72]. In addition, large-sized billfishes (Istiophoridae) were also part of the Pliocene Mediterranean fauna [73], and a conspicuous fossil record suggests that soft-shelled turtles (i.e., the trionychids, which often occur in marine waters; [74]) were also relatively common along the Mediterranean coasts [75]. Based on neontological observations [5,11,12,13,64,76,77,78,79,80], it is reasonable to hypothesise that all these forms would have represented valuable prey/scavenging items for the Mediterranean Pliocene white sharks, and the same could be said for other co-occurring marine macropredators, including the otodontids [81] and the largest carcharhinids [82]. Not least, early juvenile mysticetes were likely more common than today in the Pliocene Mediterranean Sea, which may have contained balaenid and balaenopterid calving grounds [83,84,85,86], thus providing the Mediterranean white sharks with vulnerable, energetically valuable potential prey (see e.g., Taylor et al. [87]). If, as convincingly argued by Bianucci et al. [11], the importance of mysticetes and other low trophic-level vertebrates (e.g., sirenians) as food items for Mediterranean white sharks was greater in Pliocene times than it is today, that may provide some hints to explain the slight discrepancy in trophic level that has sometimes been observed on geochemical grounds between recent and fossil specimens of *C. carcharias* [68].

As a final caveat, we note that although fishes were probably also prominent in the diet of the Pliocene Mediterranean white sharks like they are today [10], there is no way to probe this inference through taphonomy. Indeed, few fishes possess skeletal elements that are as large and robust to likely record recognisable tooth marks like those referred to above. Fish bones and scales may exceptionally get preserved as stomach contents along with shark skeletons [88,89,90,91,92]; however, no such cases have ever been reported for *C. carcharias*—at least, relative to the knowledge of the writers.

## 5. Tracing the Rise of White Sharks as the Main Mediterranean Top Predators

As already noted above, *C. carcharias* is just one of many high trophic-level predators of the Mediterranean Pliocene, which include sharks (both Carcharhiniformes, such as *Carcharhinus leucas*, *Carcharhinus longimanus* and *Galeocerdo cuvier*, and other Lamniformes, such as *C. megalodon*, *Cosmopolitodus plicatilis* and *Parotodus benedenii*) and physeteroid cetaceans (macroraptorial sperm whales) ([82,93], and the many references therein). None of the aforementioned taxa occurs in the present-day Mediterranean Sea on a regular basis, and some (i.e., *C. megalodon*, *C. plicatilis*, *P. benedenii* and the macroraptorial sperm whales) are extinct worldwide. Abundant taphonomic evidence exists that the Mediterranean Pliocene representatives of *C. leucas*, *G. cuvier* and *C. plicatilis* used to consume cetaceans both as prey and as scavenging items [36,94,95,96,97,98], whereas foraging on cetacean carrion has recently been proposed for the enigmatic, large-sized (up to more than 7 m total length; [99]) ‘false mako’ shark, *P. benedenii* [81], which most authors interpret as a formidable carnivore [100]. Thus, one may be led to think that competitive exclusion resulted in *C. carcharias* persisting in the Mediterranean Sea until the present day at the expenses of a broad array of marine macropredators. However, this is not the most parsimonious interpretation of the fossil record, which in turn indicates that many of the above taxa co-existed in the Mediterranean Basin for most of the Pliocene at least; exceptions are essentially limited to *C. megalodon* and the macroraptorial sperm whales, which are known from a handful of Zanclean finds and a single record of general Pliocene age, respectively [36,101,102]. Competitive exclusion typically includes coexistence for an ecologically brief time, a lengthy coexistence of two or more species followed by the disappearance of one being in turn suggestive of causes beyond the presence of the remaining species [103]); therefore, in the case studied here, ecological dynamics other than competitive exclusion are likely to have been at play.

In our opinion, the conspicuous climatic and environmental perturbations that affected the Mediterranean region during the last three million years or so likely played a crucial role in leading *C. carcharias* to become the main apex predator of the present-day Mediterranean waters. In particular, subsequent pulses of climate cooling that occurred from the late Piacenzian throughout the Quaternary likely led to the basin-wide extirpation of strongly thermophilic forms, both between the elasmobranch high trophic-level predators (e.g., *C. leucas*) and their potential prey (e.g., the dugongid sirenians; [72]). Variations in the productivity and temporal/spatial distribution of nutrients within the basin [82,96], as well as in the availability of shelfal environments [104], may also have resulted in periods of low prey availability and heightened extirpation risk among the marine apex predators of the Mediterranean Sea. Other disappearance events (e.g., those involving *C. hastalis* and *P. benedenii*) had a worldwide dimension, and as such, they may be better understood in a global rather than regional framework [105]. Although these extinctions/extirpations started in Pliocene times [106], they continued well into the Quaternary, with potential shark prey and scavenging items such as the right and grey whales persisting in the Mediterranean Sea until antiquity [107], and monk seal populations being brought to the brink of extinction not earlier than the last few centuries [108]. Eventually, this sequence of regional as well as global disappearances led *C. carcharias* to become the unrivalled elasmobranch apex predator of the present-day Mediterranean Sea. At the same time, it possibly favoured the radiation of another temperate, mammalophagous though essentially generalist marine predator: the killer whale, *Orcinus orca*. Indeed, the latter emerged as a macroraptorial feeder from mainly piscivorous ancestors in the subfamily Orcininae, all of which are known from the Mediterranean Pliocene [93].

All things considered, the fossil record highlights the ecological plasticity of the Mediterranean white sharks, which persisted through the fairly massive climatic and environmental perturbations that characterised the latter part of the Pliocene and the Quaternary without enjoying immigration by their North Atlantic conspecifics [22]. While the overall generalist trophic habits of white sharks [109] were likely crucial for securing ecological success in the highly variable Mediterranean scenario by allowing the transition to a more strongly piscivorous diet as the regional marine mammal fauna shrank, the role of *C. carcharias*’ endothermy [110,111,112] is unclear, as endothermic traits appear to be widespread in Lamniformes, including the extinct megatooth sharks in the family Otodontidae [113] as well as the sluggish, microphagous basking sharks in the family Cetorhinidae [114].

## 6. Concluding Remarks

### 6.1. Conclusions

We provided an updated synthesis of the palaeobiology and palaeoecology of the white shark, *C. carcharias*, in the Mediterranean Sea. The deep past of the Mediterranean population of this iconic elasmobranch species is witnessed by a rather conspicuous, mostly Pliocene fossil record. Phenetically modern white shark teeth first appeared in the Mediterranean Basin soon after the very origin of *C. carcharias*, which appears to have emerged in the Pacific Ocean around the Miocene–Pliocene transition. Colonization of the Mediterranean Sea was likely due to the dispersal of Australian/Pacific palaeopopulations through the Central American Seaway. Gigantic teeth assigned to *C. carcharias* exist in the Mediterranean Pliocene, evoking white shark individuals as large as 7 m total length. The diverse and abundant Pliocene marine mammal fauna of the Mediterranean Basin likely played a major role in sustaining the co-occurring population of *C. carcharias* through the Pliocene and most of the Quaternary. Notwithstanding the simultaneous disappearance of many potential prey items in the context of the latest Pliocene and Quaternary climatic and environmental perturbations, white sharks appear to have become increasingly more common in the Mediterranean Sea starting from the earliest. On the other hand, other macropredators have followed the opposite path, and most of them have disappeared since. The generalist feeding preferences of *C. carcharias* were probably instrumental to ensuring ecological success in the ever-changing Mediterranean setting, allowing for the transition to a more strongly piscivorous diet as the co-occurring marine mammal fauna declined.

### 6.2. Perspectives

For at least two millennia, the Pliocene and Quaternary marine deposits of the Mediterranean Basin have yielded fossil teeth of *C. carcharias*. That said, as the modern palaeontological standards privilege the study of materials whose geographic and stratigraphic whereabouts are ascertained, the scientific relevance of many of these and other finds has suffered a setback over the past few decades [49,115,116]. Hopefully, the prospection of new fossiliferous localities and the collection of white shark specimens in a stratigraphically controlled way will result in refining our understanding of the palaeobiology and palaeoecology of the Mediterranean white sharks across the last few million years.

As already noted above, the Mediterranean fossil record of *C. carcharias* is mostly Pliocene, and by and large Italian. We are not aware of any published find of *C. carcharias* from the Eastern Mediterranean Basin (Figure 3), where white sharks are known to occur at present [7]. That said, previous finds of marine vertebrates from this broad region suggest a good palaeontological potential [117], and photographs of fossil white shark teeth that have allegedly been found in Greece and Cyprus are present in some social media. Even if some of such records may have been published in local magazines, white shark fossils from along the Aegean and Levantine coasts are by and large unknown to the international scientific community. Future research efforts may be directed to fill this gap in the geographic distribution of the Mediterranean fossil occurrences of *C. carcharias*.

As the Mediterranean population of *C. carcharias* appears to have been essentially isolated since its foundation [6], intra-Mediterranean nursery areas did likely play a crucial role in recruitment from the Early Pliocene onwards. This is relevant to the present paper as, in some cases, the fossil record has the potential to disclose ancient elasmobranch nursery grounds. With respect to *C. carcharias*, a palaeo-nursery has recently been proposed to have existed near Coquimbo (Chile), based on the local occurrence of mostly juvenile white shark teeth of Pliocene age [118]. Similarly, the possible occurrence of a white shark nursery ground in the Ligurian or upper Tyrrhenian seas during the Holocene Climate Optimum was evoked by Collareta et al. [22] to explain the overall small size of *C. carcharias* teeth from the Northgrippian of Torre del Lago, in Tuscany [119]. Thus, we invite our fellow vertebrate palaeontologists to comb the Mediterranean fossil record in search of the ancient nurseries of *C. carcharias*.

As already noted elsewhere [7,21,120], a combination of slow life history, genetic isolation, negative reputation and consequent persecution, and widespread degradation of coastal habitats implies that the future of the Mediterranean white sharks is at best uncertain. Here, we would like to highlight that the fossil record has the potential to illustrate how the Mediterranean white sharks managed to survive through fairly conspicuous climatic and environmental perturbations without obvious connections with their adjacent Atlantic conspecifics. Palaeontology matters for conservation [121], and a better knowledge of the palaeobiology and palaeoecology of the Mediterranean population of *C. carcharias* may prove relevant in the current context of widespread biodiversity loss.

## Data Availability

Not applicable.
